# Functional analysis of a monoclonal antibody reactive against the C1C2 of Env obtained from a patient infected with HIV-1 CRF02_AG

**DOI:** 10.1186/s12977-021-00568-y

**Published:** 2021-08-21

**Authors:** Hasan Md Zahid, Takeo Kuwata, Shokichi Takahama, Yu Kaku, Shashwata Biswas, Kaho Matsumoto, Hirokazu Tamamura, Shuzo Matsushita

**Affiliations:** 1 Division of Clinical Retrovirology, Joint Research Center for Human Retrovirus infection, Kumamoto University, 2-2-1 Honjo, Chuo-ku, Kumamoto, 860-0811 Japan; 2 Laboratory of Immunosenescence, National Institutes of Biomedical Innovation, Health and Nutrition, Osaka, Japan; 3 Institute of Biomaterials and Bioengineering, Tokyo Medical and Dental University, Tokyo, Japan

**Keywords:** HIV-1, Non-neutralizing antibody, C1C2 antibody, ADCC, Non-subtype B, CRF02_AG

## Abstract

**Background:**

Recent data suggest the importance of non-neutralizing antibodies (nnAbs) in the development of vaccines against HIV-1 because two types of nnAbs that recognize the coreceptor binding site (CoRBS) and the C1C2 region mediate antibody-dependent cellular-cytotoxicity (ADCC) against HIV-1-infected cells. However, many studies have been conducted with nnAbs obtained from subtype B-infected individuals, with few studies in patients with non-subtype B infections.

**Results:**

We isolated a monoclonal antibody 1E5 from a CRF02_AG-infected individual and constructed two forms of antibody with constant regions of IgG1 or IgG3. The epitope of 1E5 belongs to the C1C2 of gp120, and 1E5 binds to 27 out of 35 strains (77 %) across the subtypes. The 1E5 showed strong ADCC activity, especially in the form of IgG3 in the presence of small CD4-mimetic compounds (CD4mc) and 4E9C (anti-CoRBS antibody), but did not show any neutralizing activity even against the isolates with strong binding activities. The enhancement in the binding of A32, anti-C1C2 antibody isolated from a patient with subtype B infection, was observed in the presence of 1E5 and the combination of 1E5, A32 and 4E9C mediated a strong ADCC activity.

**Conclusions:**

These results suggest that anti-C1C2 antibodies that are induced in patients with different HIV-1 subtype infections have common functional modality and may have unexpected interactions. These data may have implications for vaccine development against HIV-1.

**Graphical abstract:**

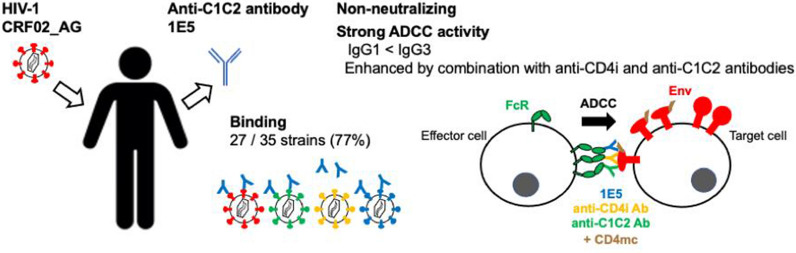

**Supplementary Information:**

The online version contains supplementary material available at 10.1186/s12977-021-00568-y.

## Background

The human immunodeficiency virus (HIV-1) envelope glycoprotein trimer (Env) is exposed on the surface of both virions and infected cells. Thus, Env is the principal target for neutralizing antibodies and antibodies able to mediate antibody-dependent cellular cytotoxicity (ADCC). HIV-1 Env is a flexible molecule that exists in at least three different conformational states: states 1, 2 and 3 [1]. Before interacting with the primary receptor, CD4, Env preferentially adopts a compact, “closed” conformation (state 1) that is largely antibody-resistant. CD4 binding “opens” Env, increasing the vulnerability of infected cells to ADCC mediated by non-neutralizing antibodies (nnAbs), as these easily-elicited antibodies preferentially recognize epitopes exposed in the open conformational states (states 2/3). These antibodies include the anti-coreceptor binding site (CoRBS) and the anti-C1C2 families of antibodies that, in combination with a small molecule that mimics CD4 (CD4mc), stabilize a new asymmetric Env conformation (state 2A) that is vulnerable to ADCC [1]. Approaches aimed at stabilizing this “open” conformation represent new interventional approaches to fight HIV-1 infection.

ADCC can play a major role in limiting the infection and replication of HIV-1 [2–5]. Data on the correlates of protection in the RV144 vaccine trial suggested that in protected vaccinees, increased ADCC activity resulted into decreased HIV-1 acquisition [6]. Antibodies binding to epitopes in the C1C2 region and mediating potent ADCC were isolated from some RV144 vaccinees [7]. Most of the nnAbs mediating ADCC require Env in a CD4-bound conformation [8] and target epitopes that overlap epitopes recognized by the anti-C1C2 antibody, such as A32 [9–11]. These CD4-induced (CD4i) immunoglobulins (IgGs) are present in the sera, breast milk and cervicovaginal lavages of HIV-1-infected patients [12, 13].

CD4mc and anti-CoRBS antibodies (Abs) bind sequentially to Env trimer, opening its conformation and allowing recognition by anti-C1C2 antibodies whose epitopes are located in the gp120 inner domain and remain occluded in the native trimer [10, 14–17]. It is not only Fab fragments that interact with the same Env trimer, but Fc fragments of these two families of Abs also bind synergistically with FcγRIIIa [18]. Fc-dependent mechanisms can impact on the viral load [19, 20] and slow disease progression by controlling HIV-1 infection [21, 22].

Recent reports have described the superior nature of the IgG3 class of antibodies over the IgG1 class, not only for Fc-receptor-mediated functions, such as ADCC and antibody-dependent cellular phagocytosis (ADCP) [21, 23–27], but also for their neutralization capability [25]. The level of IgG3 correlated with anti-HIV-1 function in the RV-144 trial [24, 27] and was involved in the control of disease in different cohorts [21, 28]. The hinge region of IgG3, which links the Fab and the Fc regions, is two to four times longer than that of IgG1. This increased hinge length may have an effect on the flexibility of the antibody and the recognition of antigen, which would ultimately result into differences in protection [29, 30]. Although IgG3 has higher functional potential, it has not been advanced clinically partly because of its shorter half-life compared with IgG1 [31, 32].

CRF02_AG comprises 46% of the circulating strains in West Africa and is the fourth most abundant subtype in the world [33]. It is also considered as the most frequent non-B subtype spreading among European natives [34]. In this study we isolated a monoclonal antibody (mAb) against the C1C2 epitope, 1E5, from a CRF02_AG-infected patient. IgG1 and IgG3 forms of 1E5 were constructed and examined for their functional characteristics. Previous studies on ADCC have focused on anti-C1C2 antibodies from subtype B-infected patients, such as A32 and C11, which are also known as anti-cluster A antibodies [11, 12, 35]. We observed the ADCC activity of 1E5 against cells expressing HIV-1 Env, although no neutralizing activity of 1E5 was detected against HIV-1. Moreover, enhancement in the binding and ADCC activities of A32 were observed in the presence of 1E5. The combination of anti-C1C2 antibodies induced by different subtypes may have implications for vaccine development against HIV-1.

## Results

### Isolation of monoclonal antibody 1E5 from a donor infected with CRF02_AG

**Fig. 1:**
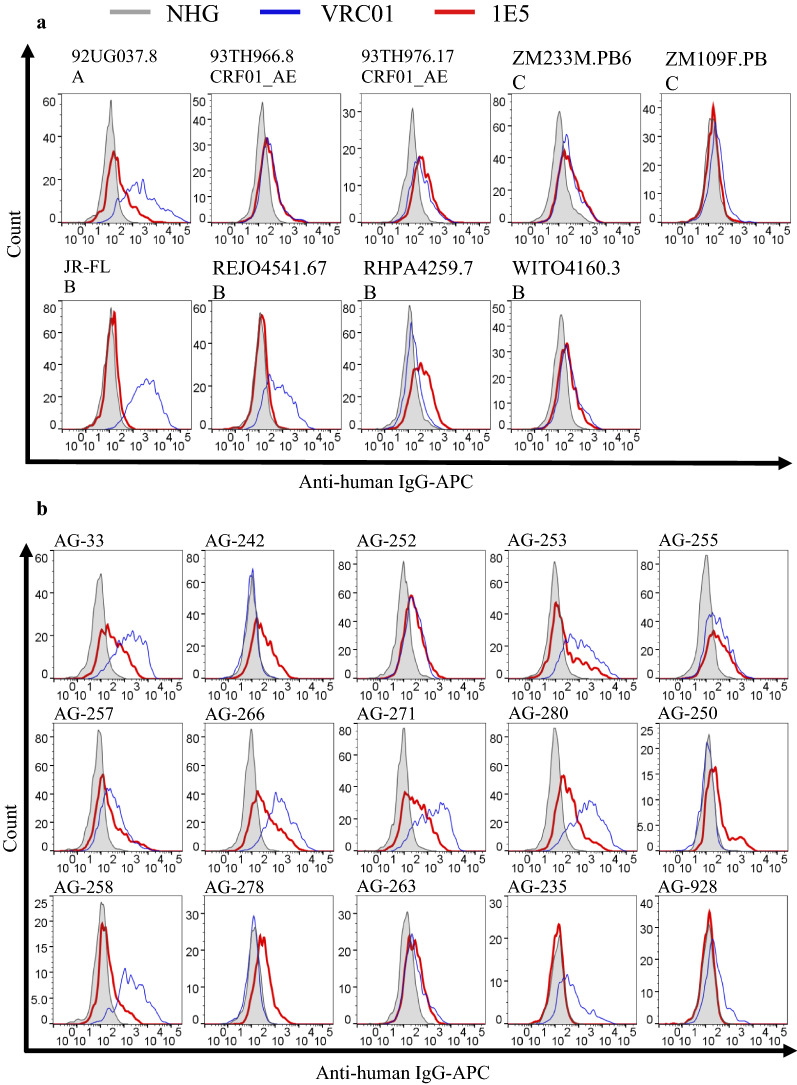
Recognition of HIV-1 Env proteins by 1E5. HEK 293T cells were transfected with plasmid expressing EGFP and Env proteins of subtypes A, AE, B and C (**a**) and those of a panel of CRF02_AG (**b**). At 48 h post-transfection, cells were stained with primary antibody. Then, cells were fluorescently labeled with an allophycocyanin (APC)-conjugated anti-human IgG secondary Ab. Histograms of APC in EGFP^+^ cells stained with 1E5, normal human globulin (NHG) as a negative control, and VRC01 as a positive control, are shown by a blue line, gray shading and a red line, respectively

B cells from a donor infected with CRF02_AG were transformed by Epstein–Barr virus (EBV) and the supernatants were screened for reactivity to the Env of HIV-1 93TH966.8 (CRF01_AE) strain. Recombinant mAb, 1E5, was isolated from the single cell-sorted Env-reactive culture by RT-PCR of the immunoglobulin heavy and light chain genes. Genetic analysis revealed that 1E5 used IGHV1-69*09 and IGKV3-20*01 as germline genes of the heavy and light chain genes, respectively (Additional file 1: Fig. S1). The binding activity of 1E5 to Env proteins from various HIV-1 strains was examined by flow cytometry (Fig. 1a). This mAb bound well to the Env protein of subtype A (92UG037.8), CRF01_AE (93TH976.17 and 93TH966.8), subtype C (ZM233M.PB6) and subtype B (WITO4160.33 and RHPA4259.7). The results suggested that 1E5 cross-reacts to HIV-1 strains belonging to various subtypes, although the Env proteins of subtype C (ZM109F.PB4) and subtype B (REJO4541.67 and JR-FL) were not recognized by 1E5. The binding activity of 1E5 to intra-subtype strains was tested using a panel of CRF02_AG Env proteins (Fig. 1b). The binding of 1E5 was observed in most CRF02_AG strains (13 out of 15 strains), suggesting that the 1E5 epitope is conserved among most CRF02_AG strains. However, the reactivity of 1E5 was moderate for AG-250, AG-258, AG-278 and AG-263, and was not detected for AG-235 and AG-928.

### IgG3 form of 1E5 showed better reactivity than the IgG1 form

**Fig. 2:**
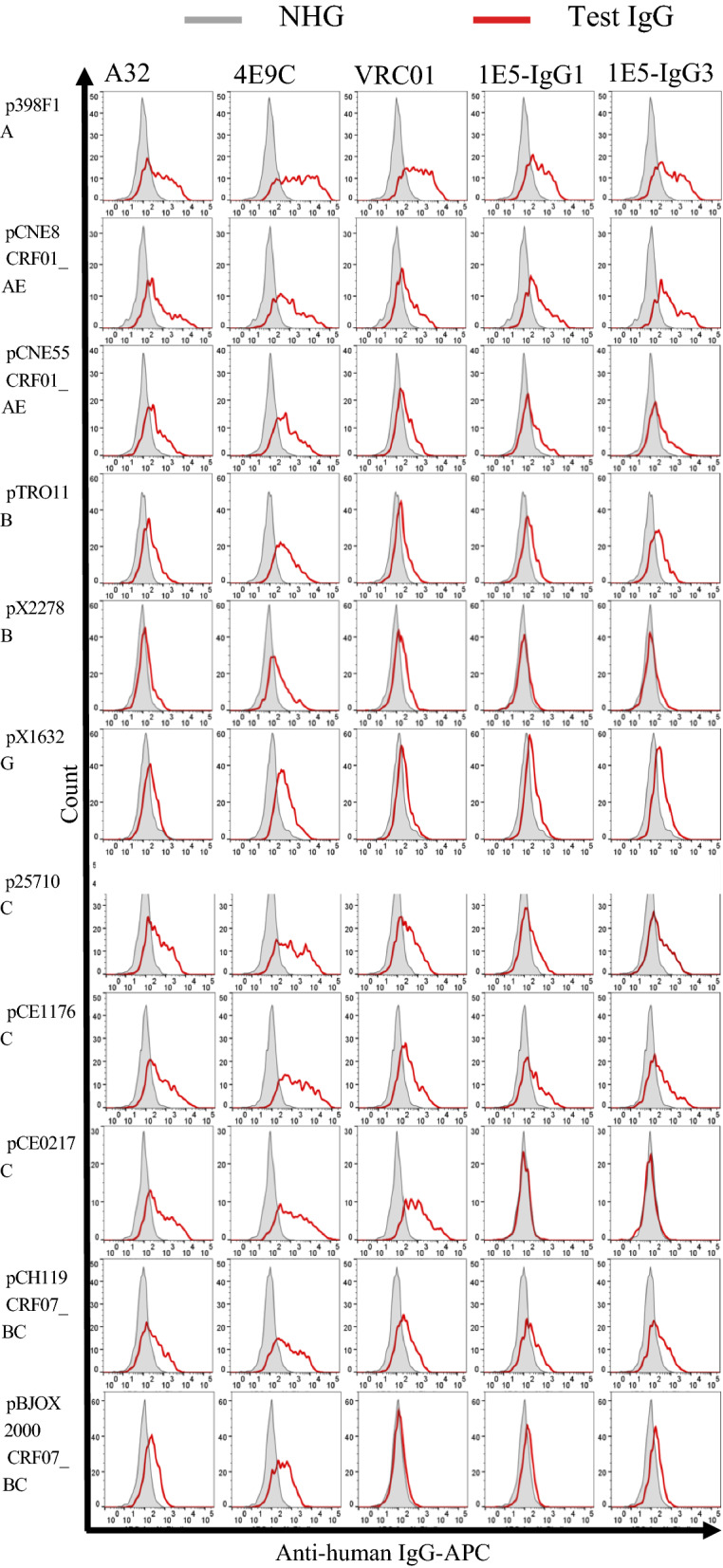
Reactivity of the IgG1 and IgG3 forms of 1E5 to global panel of HIV-1 Envs. HEK 293T cells were transiently transfected with plasmids expressing genes for both Env and EGFP. At 48 h post-transfection, cells were stained with the test IgG 1E5 (IgG1 and IgG3 forms) and three control IgGs: A32 (cluster A), VRC01 (CD4bs) and 4E9C (CoRBS). Cells were then fluorescently labeled with an APC-conjugated anti-human IgG secondary Ab. Histograms of APC in EGFP^+^ cells stained with test IgG and NHG as a negative control are shown by a red line and gray shading, respectively

Recent reports described the superior nature of the IgG3 class over IgG1 not only for Fc-receptor-mediated functions, such as ADCC and ADCP, but also for neutralization capability [21, 23–27]. To obtain 1E5 with superior activities, we constructed an IgG3 form of 1E5 in addition to the IgG1 form. Analysis of cross-reactivity using a global panel of HIV-1 [36] showed that both IgG1 and IgG3 forms of 1E5 reacted to 8 out of 11 strains (Fig. 2). The reactivity of 1E5 appeared high for subtype A and AE strains, such as p398F1 and pCNE8, as well as subtype C and BC strains, such as p25710, pCE1176 and pCH119. The 1E5 reacted moderately to pCNE55, pTRO11, pX1632 and pCH119, but showed no detectable reactivity to pX2278, pCE0217 and pBJOX2000. The reactivity of the IgG3 form of 1E5 was significantly higher than that of the IgG1 form (Additional file 2: Fig. S2). Taken together, the binding data revealed that 1E5 reacted to 27 out of 35 strains tested (77%).

### Determination of the epitope recognized by 1E5

**Fig. 3:**
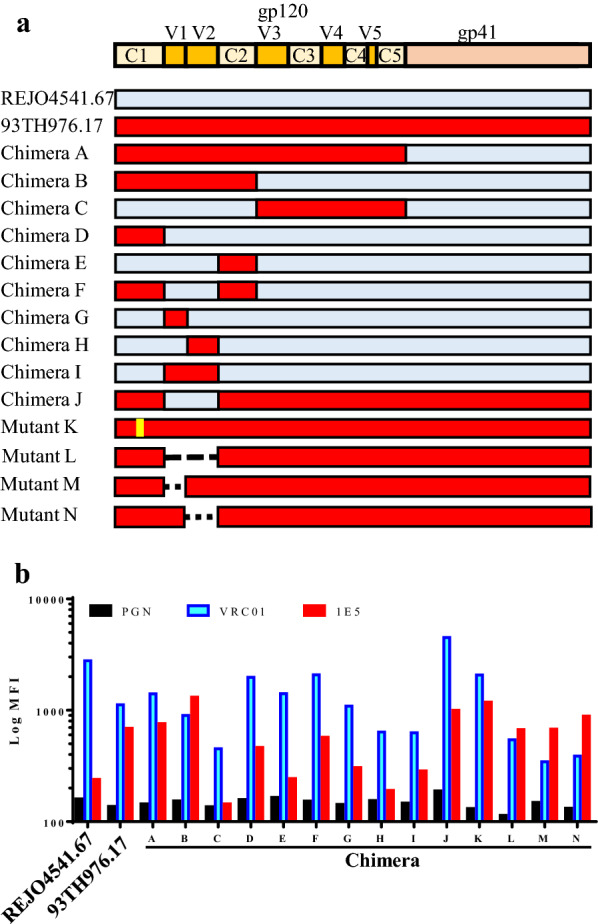
Determination of the Env regions required for 1E5 binding. **a** Schematic presentation of the Env recombinants constructed between REJO4541.67 (SVPB16), which is not recognized by 1E5, and 93TH976.17, which is strongly bound by 1E5. The regions from REJO4541.67 and 93TH976.17 are shown in light-blue and red, respectively. The W69G mutation is shown in yellow. Deletion in the V1, V2 and V1V2 region was shown by a dotted line. **b** The reactivity of Env recombinants to NHG, VRC01 and 1E5 was determined by flow cytometry analysis using cells expressing each Env recombinant. The reactivity was detected by APC-conjugated anti-human IgG secondary Ab, and the mean fluorescence intensity (MFI) of APC is shown. Experiments were performed three times, and the representative result is shown

To determine the 1E5 epitope, a panel of Env mutants, which were affected in their reactivity of potent anti-HIV-1 antibodies [37–39], were used to examine the reactivity to 1E5. However, the results revealed that point mutations in V2 (N160K, I165A, L165A, K169E, L175P and L179P), the CD4 binding site (CD4bs, D368R) and the CD4-induced epitope (CD4i, I420R) did not change the reactivity of 1E5 (Additional file 3: Fig. S3). Furthermore, the addition of sCD4 or CD4mc, YIR-821 [40], did not change the reactivity of 1E5 (Additional file 4: Fig. S4). These results suggested that the V2, CD4bs, CD4i and V3 epitopes are not the target for 1E5. Next, we compared the binding activity of 1E5 to chimeric Env proteins from 93TH976.17, a 1E5-reactive strain, and REJO4541.67, a 1E5-non-reactive strain (Fig. 3). Chimera A, which possesses gp120 from 93TH976.17 and gp41 from REJO4541.67, retained reactivity to 1E5. Chimeric Env containing the C1-C2 domains from 93TH976.17 (Chimera B) showed reactivity, but that containing the V3-C5 domains (Chimera C) resulted in no reactivity, indicating that the 1E5 epitope is in the C1-C2 domain of gp120. Strong binding was observed when the C1 and C2 domains originated from 93TH976.17 (Chimera F), although all of the chimeric Env constructs possessing the C1-C2 domain of 93TH976.17 (Chimeras D, E, G, H and I) showed marginal reactivity to 1E5. Moreover, strong binding of 1E5 to the V1/V2 deletion mutants clearly demonstrated that the epitope of 1E5 is not in the V1V2 region (Chimeras L, M and N). This suggested that the C1 and C2 domains constitute the 1E5 epitope, but that the V1 and V2 domains also affect the binding of 1E5. Consistent with this, chimera J, which contains the V1-V2 domain from REJO4541.67 in a 93TH976.17 backbone, showed good reactivity to 1E5. The data also suggested that the epitope recognized by 1E5, consisting of C1 and C2, may be different from that of A32 because the W69G mutation had no effect on binding [11, 41].

**Fig. 4:**
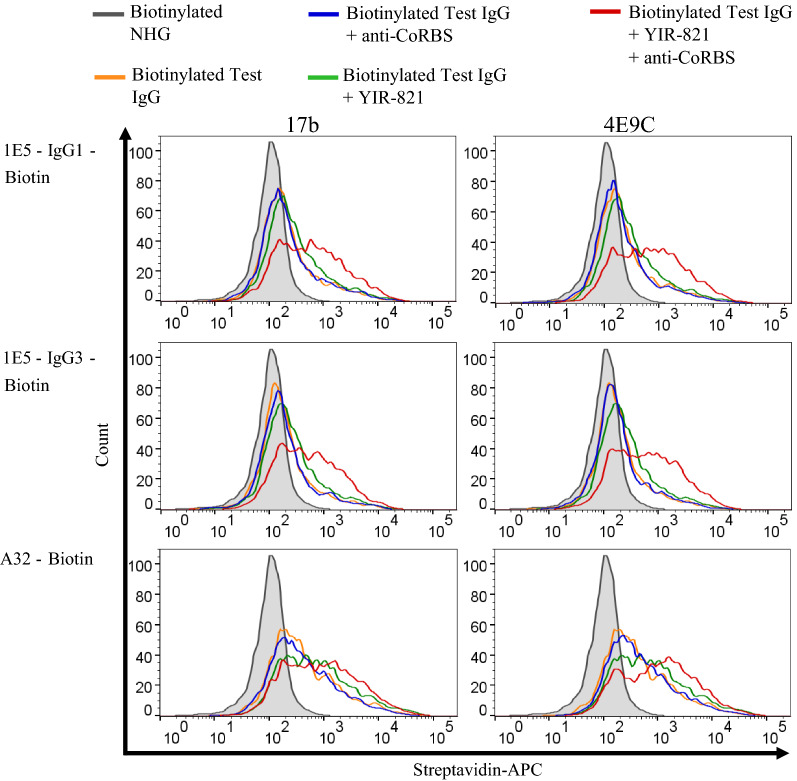
Anti-CoRBS antibody and CD4mc increased binding of 1E5 to CRF02_AG Env. HEK 293T cells were transfected with plasmid expressing both EGFP and CRF02_AG-257 Env. At 48 h post-transfection, cells were stained with biotinylated test IgG (1E5-IgG1 and 1E5-IgG3, 10 µg/ml) alone or with anti-CoRBS antibody (17B or 4E9C, 5 µg/ml) in the presence or absence of CD4mc YIR-821 (20 µM). Biotinylated A32 and biotinylated NHG were used as a control. Then, cells were fluorescently labeled with APC-conjugated streptavidin. Histograms of APC in EGFP^+^ cells are shown; Biotinylated NHG (gray shading), test IgG alone (orange), test IgG and YIR-821 (green), test IgG and anti-CoRBS antibody (blue), and IgG, YIR-821 and anti-CoRBS antibody (red). The representative result from three independent experiments is shown. Each independent experiment was done at once with the same transfected cells

The conformational epitope consisting of the C1 and C2 domains of gp120 contains the cluster A region [10, 42]. The binding of antibodies to cluster A, such as A32 and C11, was reported to be enhanced by a combination of CD4mc and anti-CoRBS antibodies [8, 11, 35]. To investigate the activity of anti-cluster A antibody, the binding activity of 1E5 was analyzed in the presence of CD4mc and anti-CoRBS antibodies (Fig. 4). The addition of CD4mc (YIR-821) and an anti-CoRBS antibody (17b or 4E9C) [43, 44] markedly enhanced the binding activity of 1E5. This enhancement required both CD4mc and anti-CoRBS antibody, and the addition of either CD4mc or anti-CoRBS antibody alone did not affect 1E5 binding significantly. This enhancement effect of CD4mc and anti-CoRBS antibody on 1E5 binding was even more apparent than that on A32 binding.

To further investigate whether 1E5 binds to an epitope that overlaps with the epitope for A32, we performed a binding inhibition assay using Env from CRF02_AG-257-transfected cells as the target and biotinylated 1E5 or A32 as the probe (Additional file 5: Fig. S5). The findings revealed that 1E5 did not compete with A32 for binding (Additional file 5: Fig. S5a), but significant enhancement of A32 binding was observed in the presence of 1E5 (Additional file 5: Fig. S5b). These data suggested that 1E5 binds to the C1C2 region that does not overlap with the A32 epitope, and further that binding of 1E5 can enhance the binding of A32.

### Neutralization and ADCC activities of 1E5

The neutralization activity of 1E5-IgG1 was tested by a standard single-round neutralization assay for HIV-1 strains belonging to subtype A, CRF01_AE and CRF02_AG (Additional file 6: Fig. S6a). The neutralization activity of 1E5-IgG3 was examined by 1E5 alone and 1E5 with anti-CoRBS antibody and CD4mc against CRF02_AG-257 virus (Additional file 6: Fig. S6b). Neither the IgG1 nor the IgG3 forms of 1E5 showed any neutralization activity, similar to the other C1C2 antibodies [9, 11, 35].

**Fig. 5:**
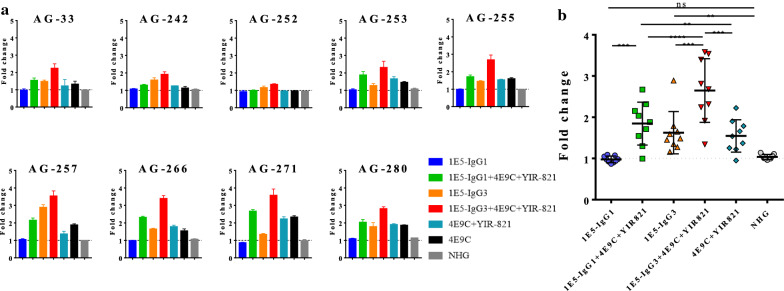
1E5-IgG3 induces higher ADCC than 1E5-IgG1 in the presence of anti-CoRBS antibody and CD4mc. The ability of IgG1 and IgG3 forms of 1E5 to mediate signaling through FcγRIIIa when bound to cells expressing Env is shown. HEK 293T cells transfected with HIV-1 Env (nine binding assay positive Env proteins from a CRF02_AG panel) were incubated with the indicated antibodies and the ADCC indicator cell line expressing FcγRIIIa. Simultaneous binding of antigen and FcγRIIIa results in activation of the NFAT transcription factor, which induces luciferase in indicator cells. IE5 was used (10 µg/ml) alone or in combination with 4E9C (5 µg/ml) and CD4mc YIR-821 (20 µM). The fold change was calculated by dividing the luminescence units in the presence of Ab with those in the absence of Ab. NHG was used as a control. **a** ADCC activity against each target cell is shown. Experiments were performed in triplicate, and the means ± standard errors of the means are shown. **b** ADCC activity against target cells expressing Env from nine CRF02_AG strains was plotted, and statistically analyzed. The means ± standard errors of the means are shown. Statistical significance was tested using a paired t test (*, P < 0.05; **, P < 0.01; ***, P < 0.001; ****, P < 0.0001; ns, non-significant)

**Fig. 6:**
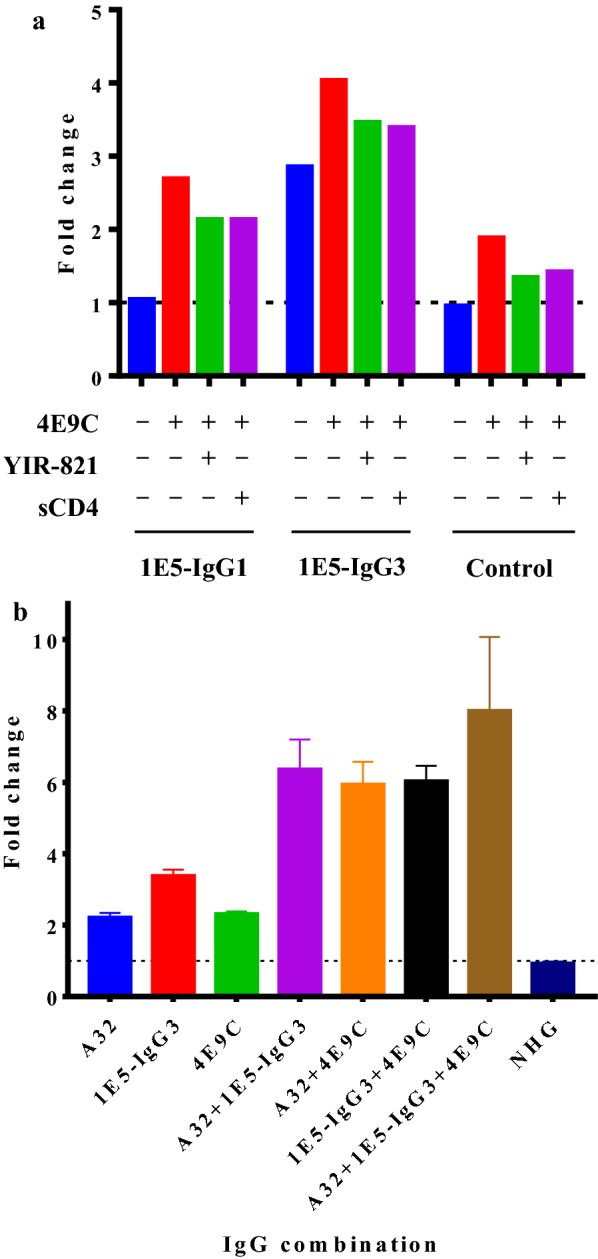
Combination of anti-C1C2 antibodies and anti-CoRBS antibody enhanced ADCC activity. The ability of 1E5 to mediate ADCC activity was analyzed by measuring the signal through FcγRIIIa. HEK 293T cells expressing AG-257 Env were incubated with the indicated antibodies, YIR-821 and sCD4, and co-cultivated with the ADCC indicator cell line expressing FcγRIIIa. Fold change was calculated by dividing the luminescence units in the presence of Ab by those in the absence of Ab. NHG was used as a control. **a** 1E5-IgG1 or 1E5-IgG3 was used (10 µg/ml) alone or in combination with 4E9C (5 µg/ml), sCD4 (2 µg/ml) or CD4mc YIR-821 (20 µM). The effect of 4E9C, sCD4 and YIR-821 in the absence of test antibody is shown as a control. **b** Anti-C1C2 antibodies, A32 and 1E5-IgG3, and anti-CoRBS antibody, 4E9C, were used at a concentration of 10 µg/ml. The combination of anti-C1C2 antibody and anti-CoRBS antibody, as well as the combination of two anti-C1C2 antibodies, mediated stronger ADCC activity than each antibody alone. The combination of two anti-C1C2 antibodies and one anti-CoRBS antibody reached maximum enhancement at an eight-fold increase

The ADCC activity of 1E5 was examined against nine CRF02_AG strains that showed strong binding of 1E5 (Fig. 1b) by the detection of FcγRIIIa signaling (Fig. 5a). Both IgG1 and IgG3 forms of 1E5 showed low ADCC activity against most of the strains, although 1E5-IgG3 showed higher ADCC activity than 1E5-IgG1 against several strains, such as AG-242, AG-257 and AG-280. The combination of 1E5, both IgG1 and IgG3 forms, with 4E9C and YIR-821 increased ADCC activity against all of the strains tested except for AG-252. This lack of ADCC enhancement against AG-252 was consistent with the lack of enhancement of binding activity to AG-252 by 4E9C and YIR-821 (Additional file 7: Fig. S7). The combination effect of ADCC activity was statistically significant, and the combination of 1E5-IgG3 with 4E9C and YIR-821 showed a significantly higher level of ADCC activity than the other combinations (Fig. 5b). This was consistent with the enhancement of binding activity of 1E5 with CD4mc and anti-CoRBS antibodies (Fig. 4) and previous reports describing the enhancement of ADCC activity by the combination of C1C2 antibody, CD4mc and anti-CoRBS antibodies [8, 11, 35]. However, additional analysis of the combination effect revealed that CD4mc and sCD4 were not required for ADCC enhancement in the ADCC assay detecting FcγRIIIa signaling (Fig. 6a). The addition of 4E9C alone increased ADCC activity more than the combination with CD4mc or sCD4. The lack of an effect with CD4mc and sCD4 may be due to the CD4 molecules expressed on the surface of effector cells, which possibly change the Env conformation accessible to anti-CoRBS antibodies. The level of dose-dependent ADCC activity of 1E5 in the presence of 4E9C was the same in both the presence and absence of YIR-821 (Additional file 8: Fig. S8). As reported previously [10, 11], anti-CoRBS antibody alone did not mediate ADCC despite its strong recognition of the target cells (Additional file 9: Fig. S9). These results suggested that 1E5 mediates ADCC in combination with CD4 and anti-CoRBS antibody.

### Enhancement of ADCC by the dual and triple combination of anti-C1C2 antibodies and an anti-CoRBS antibody

Although 1E5 recognized the C1C2 region of gp120, the epitope recognized by 1E5 did not overlap with that of A32, the representative anti-cluster A antibody (Additional file 5: Fig. S5a). The binding of A32 was even enhanced in the presence of 1E5 (Additional file 5: Fig. S5b), and enhancement of FcγRIIIa signal detecting ADCC activity was examined using three antibodies, 1E5, A32 and 4E9C (Fig. 6b). These antibodies mediated a two- to three-fold change in ADCC when used alone, but mediated a six to eight-fold change when used in combination. Moreover, a triple combination of antibodies showed the highest ADCC activity, although the difference of ADCC activities was not statistically significant. Taken together, these data suggested that not only the combination of anti-C1C2 antibody and anti-CoRBS antibody, but also two anti-cluster A antibodies coordinately, can mediate strong ADCC activity, the phenomenon that has not been reported previously.

### Strong ADCC activity against HIV-1-infected cells by combination of anti-C1C2 antibodies, an anti-CoRBS antibody and CD4mc

**Fig. 7:**
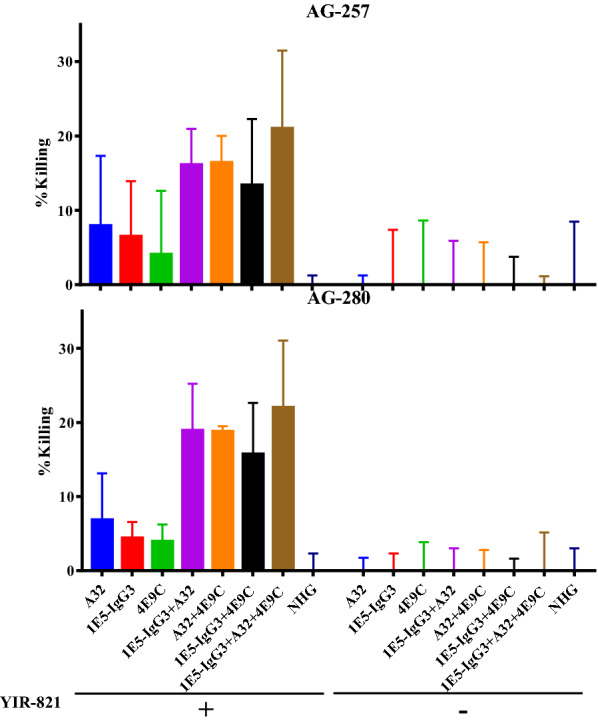
ADCC activity against HIV-1-infected cells mediated by combination of anti-C1C2 antibodies, an anti-CoRBS antibody and CD4mc. The ability of 1E5 to mediate ADCC activity was analyzed by measuring the decrease of HIV-1-infected cells. NKR24 cells infected with HIV-1, pNL-AG-257.ecto and pNL-AG-280.ecto, and N6 cells were used as target and effector cells, respectively. Decrease of HIV-1-infected cells was calculated from luciferase activity, which was increased by the LTR-driven luciferase gene in HIV-1-infected CEM.NKR-CCR5 cells. Antibodies, 1E5-IgG3 (5 µg/ml), A32 (1.25 µg/ml) and 4E9C (5 µg/ml) were used solely or in combination in the presence or absence of YIR-821 (20 µM)

We have examined ADCC activity of 1E5 by detecting FcγRIIIa signaling of the effector cells. However, this method does not measure a decrease of HIV-1-infected target cells, and expression of CD4 on the effector cell line may affect ADCC activity by changing Env conformation (Fig. 6a). To examine ADCC activity of 1E5 against HIV-1-infected cells without the effect of CD4 expression on the effector cells, we performed ADCC activity using NKR24 (CEM.NKR-CCR5 cells with LTR-Luc) cells infected with HIV-1 as target cells and N6 (human NK cell line KHYG-1 expressing FcγRIIIa) cells as effector cells. Luciferase activity, which increases by the LTR-driven luciferase gene in HIV-1-infected CEM.NKR-CCR5 cells, was measured, and the cell killing was calculated by a decrease of luciferase activity in the presence of antibodies. Decrease of infected cells was observed slightly in the presence of 1E5, A32 or 4E9C, considerably in the presence of two antibodies, and the triple combination of 1E5, A32 and 4E9C showed the strongest ADCC activity (Fig. 7). This is consistent with the results obtained by ADCC assay detecting FcγRIIIa signaling (Fig. 6b). Furthermore, YIR-821 was required for the ADCC activity mediated with these antibodies, suggesting that conformational change by CD4 is necessary for ADCC mediated with anti-C1C2 and anti-CoRBS antibodies.

## Discussion

We isolated a monoclonal antibody 1E5 belonging to the anti-C1C2 antibody family, which targets the C1C2 of HIV-1 gp120, from a patient infected with CRF02_AG. Genetic analysis of the immunoglobulin heavy (VH) and light (VL) chain variable domain gene segments revealed that VH was derived from VH1-69 and VL from the VK3-20 germline. A recent report of germline VH1-69-derived antibodies demonstrated the defining features of VH1-69-utilizing antibodies against gp120, namely: the hydrophobic nature of the complementarity determining region-2 (CDRH2) regions with grand average hydropathy (GRAVY) scores ranging from 0.34 to 2.7, shorter complementarity-determining region-3 (CDRH3) with a median CDRH3 length of 14 amino acids and a higher isoelectric point (pI) with a median value of 6.15 [45]. The characteristics of these antibodies also apply to 1E5, which has a high GRAVY score of 1.85 for the CDRH2 region, a short CDRH3 region involving 12 amino acids and a high pI value of 8.59. It has been reported that the interactions between VH1-69 CDRH2 and the cavities within HIV-1 gp120 are hydrophobic [46]. High CDRH2 hydrophobicity was detected as a unique and universal feature of the VH1-69-utilizing antibodies [45].

Binding analysis with chimeric envelope constructs indicated that 1E5 recognizes a conformational epitope involving the C1 and C2 regions of gp120 (Fig. 3). A previous detailed study mapped three unique clusters (A, B and C) of CD4i antibodies based on a cross competition assay [10]. Usually occluded Cluster A epitopes can be exposed by conformational changes mediated by cellular CD4 binding to Env trimer during the viral entry process or co-expression of CD4 and the viral envelope on the same cell surface [14, 15]. However, the Env protein expressed on the cell surface was recognized by A32 and 1E5 (Fig. 2). The reactivity of cluster A antibodies to Env trimers may be due to the HIV-1 strains used in this study. HIV-1 strains including non-B subtypes may be structurally different from the representative subtype B strains. It is also possible that the unprocessed Env protein may be the target for A32 and 1E5. A32 and C11 are the major examples of anti-cluster A antibodies possessing non-overlapping epitopes involving the C1 and C2 domains [10, 42]. A32-like antibodies were found to be associated with the majority of ADCC activity in chronically-infected patients [9]. A flow cytometry-based inhibition assay demonstrated that the epitope of 1E5 did not overlap with that of A32, and that 1E5 even enhanced A32 binding (Additional file 5: Fig. S5). Taken together, these data suggested that 1E5 binds to a part of the C1C2 region that does not overlap with the A32 epitope, but that binding of 1E5 can enhance the accessibility of A32 binding.

Despite having high polymorphism [25] and a shorter half-life than IgG1 [32], IgG3 is the most poly-functional IgG subclass, having the most potent Fc effector function covering the widest range [23]. In the RV144 HIV vaccine trial, IgG3-mediated Fc effector functions, such as ADCC, ADCP and complement deposition, correlated with protection [24, 27]. Considering these facts, the IgG3 form of 1E5 was constructed and used in different assays in parallel with IgG1. When comparing the binding activity to Env proteins from a global panel of HIV-1, the IgG3 form showed significantly stronger binding than IgG1 (Additional file 2: Fig. S2). As shown in Figs. 5 and 6, the IgG3 form of 1E5 exhibited significantly higher ADCC activity than IgG1 in any combination with CD4mc and/or anti-CoRBS antibody. Factors other than the epitope, such as the angle of binding, can influence the Fc function of an antibody [45]. Increased hinge length can allow more flexibility and therefore may increase the Fc-mediated effector functions of IgG3 [25]. Most importantly, IgG3 has the highest affinity to FcγRIIIa [26, 47]. Stronger binding of FcγRIIIa at an appropriate angle can favor the IgG3 form to mediate better ADCC than IgG1.

The binding of biotinylated 1E5 with CRF02_AG Env-expressing target cells was markedly increased in the presence of CD4mc (YIR-821) and anti-CoRBS antibody (4E9C). CD4mc and anti-CoRBS antibody alone could not mediate noticeable binding enhancement (Fig. 4). A similar pattern of binding was observed with A32. This indicates that 1E5 reactivity was the same as that of A32, requiring CD4mc and anti-CoRBS antibodies for enhanced binding and the stabilization of state 2 A in the presence of CRF02_AG-257 Env. Most of the previous studies analyzing anti-C1C2 antibody binding used Env belonging to subtype B viruses [1, 35, 48]. Here, we observed the same phenomena for CRF02_AG Env by means of anti-C1C2 antibodies 1E5 and A32.

The 1E5 did not demonstrate any neutralization activity (Additional file 6: Fig. S6) when analyzing its ability to reduce the infectivity of the subtype-A, CRF01_AE and CRF02_AG Env pseudotype viruses. Being derived from germline VH1-69, this observation indicates the ADCC potential of 1E5, as described by previous research [45]. A model was described for the sequential opening of trimeric Env that required anti-CoRBS antibodies to reveal the occluded epitope recognized by anti-C1C2 antibodies [35]. Engagement of CD4mc with the Phe43 cavity of the CD4 binding site causes a partial opening of trimeric Env, which enable anti-CoRBS antibodies to bind to Env but does not expose the inner region consisting of C1 and C2 regions. Binding of anti-CoRBS antibodies with two gp120 subunits possibly exposes epitopes recognized by anti-C1C2 antibodies resulting in state 2 A stabilization [1]. This recognition translated into efficient ADCC by anti-C1C2 antibodies [35] and may be involved in the ADCC exhibited by anti-C1C2 antibodies in HIV + sera [10, 11]. Figures 5 and 6a indicate that the highest level of ADCC was exhibited by the combination of IgG3 form 1E5 and anti-CoRBS antibody 4E9C. CD4mc (YIR-821) did not contribute to the enhancement of ADCC activity in the FcγRIIIa signaling assay, but was mandatory in the cell killing assay using HIV-1-infected cells. The lack of requirement for CD4mc in the FcγRIIIa signaling assay is explained by the expression of CD4 on the surface of effector cells used for the ADCC assay.

Several studies have suggested that ADCC may play a role in controlling HIV-1 infection [22, 49]. In the RV144 trial, ADCC was mainly found to be responsible for conferring protection [6]. The C1C2 region is immunodominant in the case of both natural infection and vaccination. The majority of the ALVAC-HIV/AIDSVAX B/E vaccine recipients developed ADCC-mediating antibodies with the C1C2 region specific A32-like antibodies comprising the significant portion [7]. This study demonstrated that the binding of A32 increased in the presence of 1E5 (Additional file 5: Fig. S5b). This observation raised the possibility of enhancement of ADCC using a combination of two anti-C1C2 antibodies. When used in an ADCC assay using FcγRIIIa signaling, the combination of 1E5-IgG3 and A32 showed higher level of fold change than their individual combinations with anti-CoRBS IgG (Fig. 6b). This combination effect by two anti-C1C2 antibodies were also confirmed in an ADCC assay using infected cells as target cells (Fig. 7). As anti-C1C2 antibodies are the mediators of ADCC activity exhibited by HIV-1 + sera [9–11], and these antibodies can be elicited by vaccination [7], targeting this combination of anti-C1C2 antibodies may be a major tool for the protection against HIV-1. Moreover, a recent study on the elicitation of anti-C1C2 and anti-CoRBS antibodies observed higher and more efficient induction of anti-C1C2 antibodies in immunized guinea pigs [50]. Our results suggested that some of the anti-C1C2 antibodies, such as 1E5, can stabilize the Env conformation at state 2a in the presence of CD4mc resulting in the enhancement of binding of the other anti-C1C2 antibodies, such as A32, to exert higher ADCC activities. This finding may have implications in terms of vaccine strategies to induce appropriate combinations of antibodies for improved outcomes.

## Conclusions

Our findings indicate that the IgG3 form of anti-C1C2 antibody 1E5 isolated from a CRF02_AG-infected individual can mediate higher ADCC than the IgG1 form. The combination of two anti-cluster A antibodies, together with an anti-CoRBS antibody, mediated the highest level of ADCC.

## Methods

### Cells and reagents

The 293T, 293 A and TZM-bl cells were maintained in Dulbecco’s modified Eagle’s medium (DMEM; Nacalai Tesque, Inc., Kyoto, Japan) supplemented with 10% heat-inactivated fetal bovine serum (FBS; Mediatech Inc., Corning, Manassas, VA, USA). Mab 4E9C [43, 44] and CD4mc YIR-821 [40] have previously been reported. Soluble CD4 was purchased commercially (sCD4, R&D systems, Inc., Minneapolis, MN, USA). Heavy and light chain gene-expressing plasmids of VRC01 [51], Env clones from a global panel [36], subtype B [52], A [52], C [53] and CRF02_AG [54] were obtained through the NIH AIDS Reagent Program. NKR24 cells, CEM.NKR-CCR5 cells with LTR-Luc, were maintained in RPMI1640 (Fujifilm, Osaka, Japan) supplemented with 10% FBS, 2mM Glutamax, 0.1 mg/ml Primocin (R10). N6 cells, human NK cell line KHYG-1 expressing human CD16, were maintained in R10 medium supplemented with 1 µg/ml Cs-A and 5 U/ml IL-2. NKR24 cells and N6 cells were kindly provided by Dr. Evans [55].

### Isolation of IgG-producing single B cells by fluorescence activated cell sorting

A blood sample was obtained from patient KMCB2 of Kyushu Medical Center, who was infected with the CRF02_AG subtype of HIV-1. B cells were transformed by EBV and cultured at a concentration of 10^3^ cells/well for 10 days, as previously reported [45]. Single cells were sorted from the wells of an EBV-transformed B cell culture that scored positive for binding to Env (HIV-1 93TH966.8)-expressing cells using FACSAria II (BD Biosciences, San Jose, CA, USA). The cells were stained with anti-human IgG-BV421 and anti-human IgM-APC/Cy7 (BioLegend, San Diego, CA, USA), and IgG^+^IgM^˗^ cells were sorted at single cell density into 4 µl/well of ice-cold 0.5× phosphate-buffered saline (PBS) containing 10 mM DTT, 8 U RNAsin® (Promega, WI, USA), 0.4 U 5′-3′ Prime RNAse Inhibitor™ (Eppendorf) as previously described [56].

### Cloning and analysis of 1E5 immunoglobulin variable genes

cDNA was synthesized as previously described [56] in a total volume of 14 µl/well in a 96-well sorting plate. Total RNA from single cells was reverse transcribed in nuclease-free water (Eppendorf) using 150 ng random hexamer primer (pd(N) 6, GE Healthcare, Buckinghamshire, UK), 0.5 µl (10 mM) of each nucleotide dNTP-Mix (Invitrogen, Carlsbad CA, USA), 1 µl (0.1 M) of DTT (Invitrogen), 0.5% v/v Igepal CA-630 (Sigma), 4 U RNAsin® (Promega), 6 U Prime RNAse Inhibitor™ (Eppendorf) and 50 U Superscript® III reverse transcriptase (Thermo Fisher Scientific, MA USA). The reverse transcription (RT) was performed as follows: 42 °C for 10 min, 25 °C for 10 min, 50 °C for 60 min and 94 °C for 5 min.

For cloning of 1E5 immunoglobulin variable genes, the first round of nested PCR was performed according to the methods described by Tiller et al. [56] using the same primer pairs, while second-round primers were modified to have a 15 base overlap at the 5ʹ end with the specific vectors. The second PCR primer sequences are listed in Additional file 10: Table S1.

The IgG heavy and light chain expression plasmids were constructed by recombination of the designated second PCR product with pIgGH and pKVA2, respectively [43], using the GeneArt Seamless Cloning and Assembly kit (Invitrogen). The nucleotide sequences of the immunoglobulin variable regions were aligned and compared to avoid possible PCR error. The sequences were analyzed for germline gene verification, framework and CDR mapping, quantification of percent identity to germline, CDR amino acid length and pI using IMGT vquest (http://imgt.org/IMGT_vquest/vquest). CDRH2 grand average of hydropathy (GRAVY) scores were calculated using an online tool (http://www.gravy-calculator.de/).

### Construction of IgG3 heavy chain-expressing plasmid

The region from CH1 to CH3 of IgG1 heavy chain-expressing vector pIgGH was exchanged with the corresponding region of IgG3, and IgG3 heavy chain-expressing vector pIgG3H was constructed. Briefly, the CH1-CH3 region of IgG3 was amplified using primers, CHApa-F (AGC CTC CAC CAA GGG CCC ATC GG), IgG3-R (TCA CCA AGT GGG GTT TTG AGC TCA), CHPme-R (CTG ATC AGC GGG TTT AAA CTA TCA TTT ACC CGG AGA CAG GG) and IgG3-F (ACA AGA GAG TTG AGC TCA AAA CCC C) from cDNA, which was synthesized from the RNA of healthy donor peripheral blood mononuclear cells. The CH1-CH3 region of pIgGH was excluded by digestion with *Apa*I and *Pme*I, and the IgG3 fragments were inserted into the vector using the GeneArt Seamless Cloning and Assembly kit (Invitrogen). The variable region of 1E5 was inserted into pIgG3H to obtain 1E5-IgG3.

### Production and purification of recombinant IgG

Recombinant IgG was produced and purified as previously described [43]. Briefly, heavy and light chain plasmids were transfected into 293 A cells using TransIT®-LT1 Transfection Reagent (Mirus Bio LLC, WI, USA), and the cells stably expressing IgG were selected with G418 (1000 µg/ml) and hygromycin (150 µg/ml). IgG1 and IgG3 proteins were purified using a HiTrap™ rProtein A FF Column and a HiTrap™ Protein G HP column, respectively (GE Healthcare).

### Analysis of the binding activity of antibodies by flow cytometry

The binding activity of antibodies was analyzed as previously described [57]. Briefly, 293T cells were transfected with a plasmid expressing both HIV-1 Env and enhanced green fluorescent protein (EGFP). After 48 h of transfection, the cells were stained with primary antibody for 15 min at room temperature (RT). The cells were washed twice with PBS containing 0.2% BSA, and incubated with allophycocyanin-conjugated AffiniPure F(ab’)2 Fragment Goat Anti-Human IgG (H + L) (Jackson ImmunoResearch, West Grove, PA, USA) for 15 min at RT. Cells were fixed with PBS containing 10% formalin and analyzed using the FACSCanto II (BD Biosciences, San Jose, CA, USA). The reactivity of the antibodies was analyzed after gating the EGFP + cells using FlowJo (TreeStar, San Carlos, CA, USA). All experiments were performed at least twice independently, and the representative results are shown.

### Neutralization assay using pseudovirus

The neutralization activity of antibodies was determined as previously described [43, 58]. In brief, 293T cells were transfected with pSG3ΔEnv and Env expression vector, and the supernatant after 48 h of transfection was stored at ˗80 °C. The median tissue culture infectious dose (TCID_50_) of each pseudovirus was determined using TZM-bl cells. Serially diluted antibody and virus (400 TCID_50_) were incubated for 1 h, and TZM-bl cells were added. After incubation for 48 h, the galactosidase activity was measured using galactosidase substrate (Tropix Gal-Screen substrate, Applied Biosystems) and an EnSpire Multimode Plate Reader (PerkinElmer, MA, USA). The relative light units (RLU) were compared to calculate the reduction in infectivity and 50% of the maximal inhibitory concentration (IC_50_) was calculated using nonlinear regression.

### ADCC reporter assay to detect FcγRIIIa-mediated signaling

The detection of FcγRIIIa-mediated signaling was performed using a Jurkat NFAT-luc FCγRIIIa cell line (BPS Bioscience, CA, USA), as described previously [59]. The target cells were 293T cells expressing Env, which were transfected with Env-expressing plasmid 48 h before the ADCC assay. The target cells were washed with PBS, treated with 0.05% trypsin, and resuspended in RPMI-1640 with 4% FBS at a concentration of 3 × 10^6^ cells/ml. Then, 25 µl of the target cells were incubated with antibodies for 15 min, after which 25 µl of effector Jurkat cells were added at a ratio of 1:1 and were co-cultured for 6 h. The cells were lysed and the firefly luciferase activity was determined with a luciferase assay kit (Promega) and EnSpire® Multimode Plate Reader. The co-culture in the absence of antibody provided background (antibody-independent) luciferase activity. The RLU obtained in the presence of antibody were divided by the background level to calculate the fold change.

### Infectious molecular clones for ADCC assay

Replication competent infectious molecular clones (IMC) were designed to encode AG-257 and 280 *env* genes in pNL4-3 (GenBank: AF324493). The region of the *env* gene that encode the ectodomain were amplified by primers, NL-4-AG-257-F (AAGCAGTAAGTAGTAAATGCAACCTTTAGC) and NL-257-ECTO-R (TATCATTATGAATAATTTTATATACCACAG) for AG-257, and NL-4-AG-280-F (AAGCAGTAAGTAGTAGATGCAATCTTTAAT) and NL-280-ECTO-R (TATCATTATGAATAATTTAATATACCACAG) for AG-280. The amplified fragment was replaced with the corresponding region of pNL4-3, generating pNL-AG-257.ecto and pNL-AG-280.ecto [60] using GeneArt Seamless Cloning and Assembly enzyme mix (Invitrogen, Carlsbad, CA, USA).

### ADCC assay using HIV-infected cells and N6 cells

ADCC assay was performed according to the method described before with some modifications [55]. In brief, NKR24 cells were infected by spinoculation in round bottom tubes. 5 × 10^5^ target cells and infectious viral inoculum were subjected to centrifugation at 1200×g for 2 h at 25 °C. Then the viral inoculum was removed, and the target cells were cultured in R10 medium for 2 days. Target cells were washed 3 times in R10 medium and suspended in R10 medium containing 10 U/ml IL-2 without CsA. In round-bottom, tissue culture-treated polystyrene 96-well plates, 40 µl each of target and effector cells were added at 2.5 × 10^5^ cells/ml and 2.5 × 10^6^ cells/ml, respectively. N6 effector cells and uninfected target cells were a control to define 0% RLU. N6 cells and infected targets without antibody were a control to define 100 % RLU. Antibodies (20 µl) were added in triplicate. After 8 h incubation, a 40 µl of cells was resuspended and mixed with 40 µl of BriteLite Plus (Perkin Elmer) in 96-well white 1/2 area microplate. Luciferase activity was measured using an EnSpire Multimode Plate Reader, and the %killing of HIV-infected cells was calculated from the decrease of RLU.

## Supplementary Information

**Additional file 1: Fig. S1.** Amino acid sequences of 1E5 heavy and light chains

**Additional file 2: Fig. S2.** gG3 exhibits stronger binding affinity to a panel of global HIV-1 Env clones. HEK 293T cells were transfected with plasmids expressing 11 Env proteins from a global panel of HIV-1. At 48 h post-transfection, cells were stained with primary antibody (1E5). Then, cells were fluorescently labeled with an APC-conjugated anti-human IgG secondary Ab. The plasmids used for transfection express HIV-1 Env and EGFP. Means ± standard errors of the means of APC intensity in EGFP^+^ cells are shown. Statistical significance was tested using a paired t test (*, P <0.05).

**Additional file 3: Fig. S3.** 1E5 did not recognize a previously well-defined epitope. HEK 293T cells were transfected with plasmids encoding both EGFP and HIV-1 Env (subtype A and CRF01_AE) with point mutations at previously described positions in V2, CD4i, CD4bs and glycosylation sites. Cells were stained with 1E5-IgG or VRC01, and binding was detected by an APC-conjugated anti-human IgG secondary Ab. The MFI values of APC in EGFP+ cells are shown.

**Additional file 4: Fig. S4.** Binding of 1E5 was not enhanced by sCD4 or CD4mc. HEK 293T cells expressing EGFP and HIV-1 Env of CRF01_AG, CRF02_AE and subtype B were incubated with sCD4 (2 µg/ml) or YIR-821 (10 µM), and stained with primary antibody (5 µg/ml). Then, IgG binding was detected by APC-conjugated anti-human IgG secondary Ab. The MFI values of APC in EGFP+ cells are shown.

**Additional file 5: Fig. S5.** Analysis of the competitive binding between 1E5 and A32. HEK 293T cells expressing EGFP and AG-257 Env were stained with 3 µg/ml of biotinylated 1E5 (a) or A32 (b) in the presence of serially-diluted non-biotinylated competitors, A32 and 1E5, respectively. Biotinylated antibodies were detected by APC-conjugated streptavidin. The MFI values of APC in EGFP+ cells are shown. The MFI value in the absence of competitor is shown by dotted line.

**Additional file 6: Fig. S6.** 1E5 is a non-neutralizing antibody. (a) The neutralization activity of 1E5-IgG1 was tested in TZM-bl assays against Env-pseudotyped viruses from subtype A, CRF01_AE and CRF02_AG of HIV-1. (b) Neutralization activity of 1E5-IgG3 was examined alone or with anti-CoRBS Ab (4E9C) and CD4mc (YIR-821) against pseudotyped virus with AG-257 Env.

**Additional file 7: Fig. S7.** Anti-CoRBS antibodies failed to increase the binding of anti-cluster A antibodies to AG-252 Env. HEK 293T cells expressing both EGFP and AG-252 Env were stained with biotinylated test IgG (1E5-IgG1, 1E5-IgG3 and A32 at 10 µg/ml) alone or with anti-CoRBS antibody (17b or 4E9C, 5 µg/ml) and CD4mc YIR-821 (20 µM), and the binding was detected by APC-conjugated streptavidin. Histograms of APC in EGFP+ cells are shown.

**Additional file 8: Fig. S8.** IgG3 form of 1E5 shows stronger ADCC in the presence of anti-CoRBS antibody. The effect of anti-CoRBS antibody and CD4mc on ADCC activity, which was measured by signaling through FcγRIIIa, was analyzed using IgG3 forms of 1E5. HEK 293T cells transfected with plasmid expressing HIV-1 Env (nine binding assay-positive Env proteins from a CRF02_AG panel and one subtype A) were incubated with the indicated antibodies and co-cultivated with the ADCC indicator cell line expressing FcγRIIIa. ADCC activities obtained by serial dilution of 1E5 (0.1, 1 and 10 µg/ml) alone (RPMI) or in combination with 4E9C (5 μg/ml) and YIR-821 (20 µM) are shown. ‘0’ on the X axis indicates the absence of 1E5. The fold change was calculated by dividing the luminescence units in the presence of Ab by those in the absence of Ab. Experiments were performed in triplicate, and the means ± standard errors of the means are shown.

**Additional file 9: Fig. S9.** Recognition of target cells by anti-CoRBS antibodies. HEK 293T cells expressing both EGFP and AG-257 Env were stained with test IgG (4E9C and 17b, 10 µg/ml) alone or in the presence of CD4mc YIR-821 (20 µM). Antibody binding was detected by APC-conjugated anti-human IgG secondary Ab. Histograms of APC in EGFP+ cells are shown

**Additional file 10: Table S1.** Second-round PCR primers.

## Data Availability

Not applicable.

## Abbreviations

CoRBS: Coreceptor binding site
ADCC: Antibody-dependent cellular-cytotoxicity
CD4mc: CD4-mimetic compounds
HIV-1: Human immunodeficiency virus type 1
Env: Envelope glycoprotein
nnAbs: Non-neutralizing antibodies
CD4i: CD4-induced
IgG: Immunoglobulins
Ab: Antibody
ADCP: Antibody-dependent cellular phagocytosis
mAb: Monoclonal antibody
EBV: Epstein–Barr virus
CD4bs: CD4 binding site
VH: Immunoglobulin heavy chain variable domain
VL: Immunoglobulin light chain variable domain
CDRH2: Heavy chain complementarity determining region-2
CDRH3: Heavy chain complementarity determining region-3
GRAVY: Grand average hydropathy
pI: Isoelectric point
sCD4: Soluble CD4
EGFP: Enhanced green fluorescent protein
TCID_50_: Median tissue culture infectious dose
RLU: Relative light units
IC_50_: 50% of the maximal inhibitory concentration
