# Supplementary material for: Functional analysis of a monoclonal antibody reactive against the C1C2 of Env obtained from a patient infected with HIV-1 CRF02_AG
Source: Retrovirology. 2021 Aug 21;18:23. doi: 10.1186/s12977-021-00568-y (PMC8379604; doi:10.1186/s12977-021-00568-y)
Supplement: Supplementary file 1 — Additional file 1: Fig. S1. Amino acid sequences of 1E5 heavy and light chains [file 12977_2021_568_MOESM1_ESM.pdf]

1E5 VH

FR1 CDR1

MDWTWRILLLVAAATGVHSQVQLVESGAEVKKPGSSVKVSCKAS GGSFTSYA INWVR

FR2 CDR2 FR3

QAPGQGLEWMGR IIPILDVP NYAQKFQGRLTIIADKSTTAAYMELSSLRSEDTAVYY

CDR3

C ARLGGSSIAFDL

1E5 VK

FR1 CDR1

MVLQTQVFISLLLWISGAYGDIVMTQSPGTLSLSRGERATLSCRAS QSVGSNFL AWY

FR2 CDR2 FR3

QQKPGQAPRLLIS AAS SRATGIPDRFSGSGSGTEFSLTISRLEPEDFAMYYC QQYG

CDR3

SSLFT
