# Supplementary figures and images for: Functional analysis of a monoclonal antibody reactive against the C1C2 of Env obtained from a patient infected with HIV-1 CRF02_AG
Source: Retrovirology. 2021 Aug 21;18:23. doi: 10.1186/s12977-021-00568-y (PMC8379604; doi:10.1186/s12977-021-00568-y)

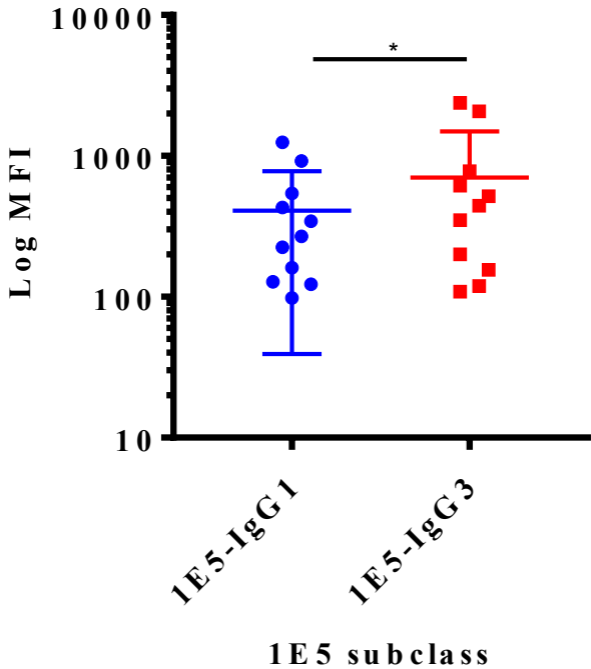

Supplement: Supplementary file 2 — Additional file 2: Fig. S2. gG3 exhibits stronger binding affinity to a panel of global HIV-1 Env clones. HEK 293T cells were transfected with plasmids expressing 11 Env proteins from a global panel of HIV-1. At 48 h post-transfection, cells were stained with primary antibody (1E5). Then, cells were fluorescently labeled with an APC-conjugated anti-human IgG secondary Ab. The plasmids used for transfection express HIV-1 Env and EGFP. Means ± standard errors of the means of APC intensity in EGFP+ cells are shown. Statistical significance was tested using a paired t test (*, P <0.05). [file 12977_2021_568_MOESM2_ESM.pdf]

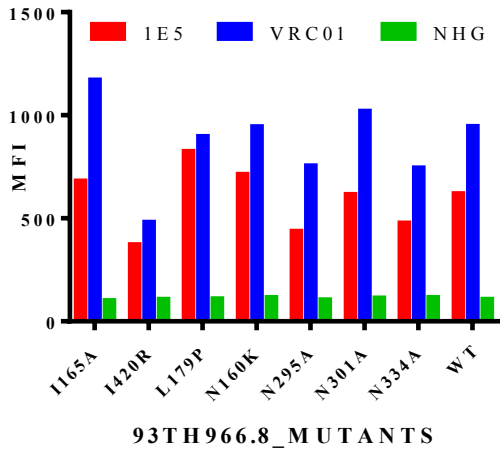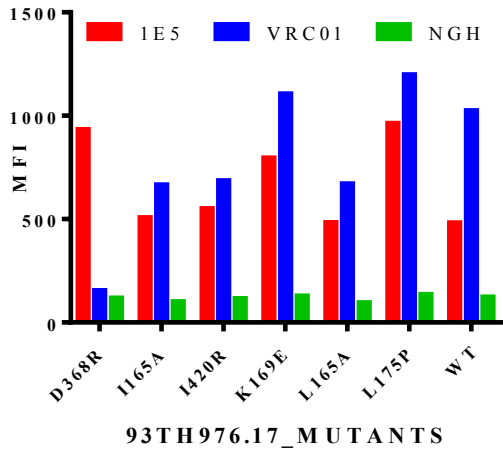

Supplement: Supplementary file 3 — Additional file 3: Fig. S3. 1E5 did not recognize a previously well-defined epitope. HEK 293T cells were transfected with plasmids encoding both EGFP and HIV-1 Env (subtype A and CRF01_AE) with point mutations at previously described positions in V2, CD4i, CD4bs and glycosylation sites. Cells were stained with 1E5-IgG or VRC01, and binding was detected by an APC-conjugated anti-human IgG secondary Ab. The MFI values of APC in EGFP+ cells are shown. [file 12977_2021_568_MOESM3_ESM.pdf]

93TH976.17

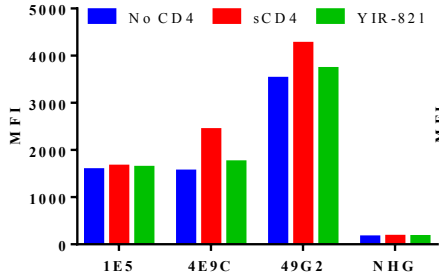

CRF02\_AG-257

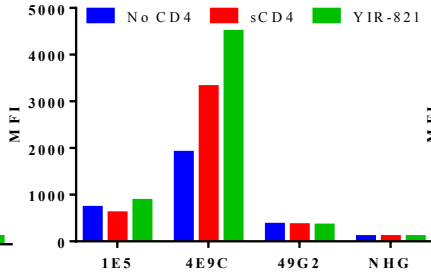

JR-FL

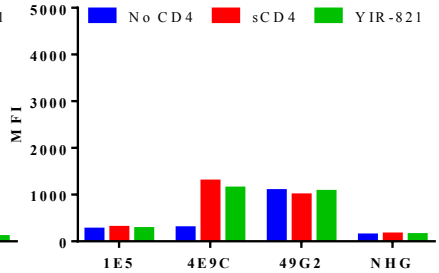

Supplement: Supplementary file 4 — Additional file 4: Fig. S4. Binding of 1E5 was not enhanced by sCD4 or CD4mc. HEK 293T cells expressing EGFP and HIV-1 Env of CRF01_AG, CRF02_AE and subtype B were incubated with sCD4 (2 µg/ml) or YIR-821 (10 µM), and stained with primary antibody (5 µg/ml). Then, IgG binding was detected by APC-conjugated anti-human IgG secondary Ab. The MFI values of APC in EGFP+ cells are shown. [file 12977_2021_568_MOESM4_ESM.pdf]

## Slide 1
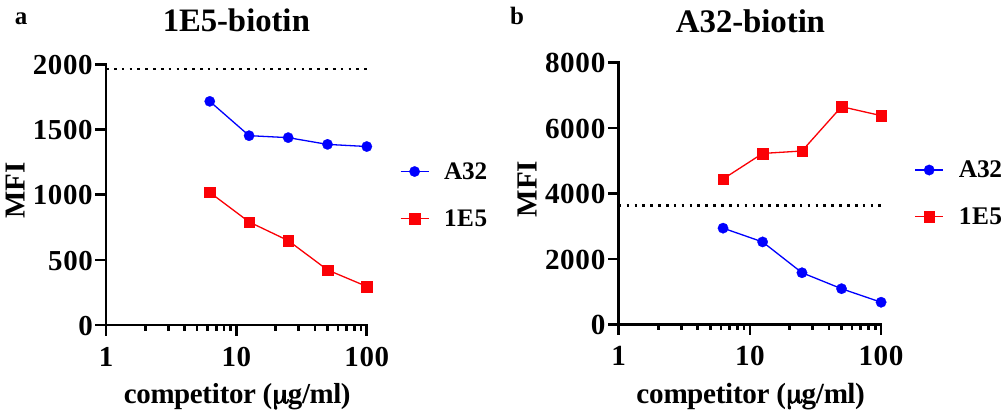

b
a

Supplement: Supplementary file 5 — Additional file 5: Fig. S5. Analysis of the competitive binding between 1E5 and A32. HEK 293T cells expressing EGFP and AG-257 Env were stained with 3 µg/ml of biotinylated 1E5 (a) or A32 (b) in the presence of serially-diluted non-biotinylated competitors, A32 and 1E5, respectively. Biotinylated antibodies were detected by APC-conjugated streptavidin. The MFI values of APC in EGFP+ cells are shown. The MFI value in the absence of competitor is shown by dotted line. [file 12977_2021_568_MOESM5_ESM.pptx]

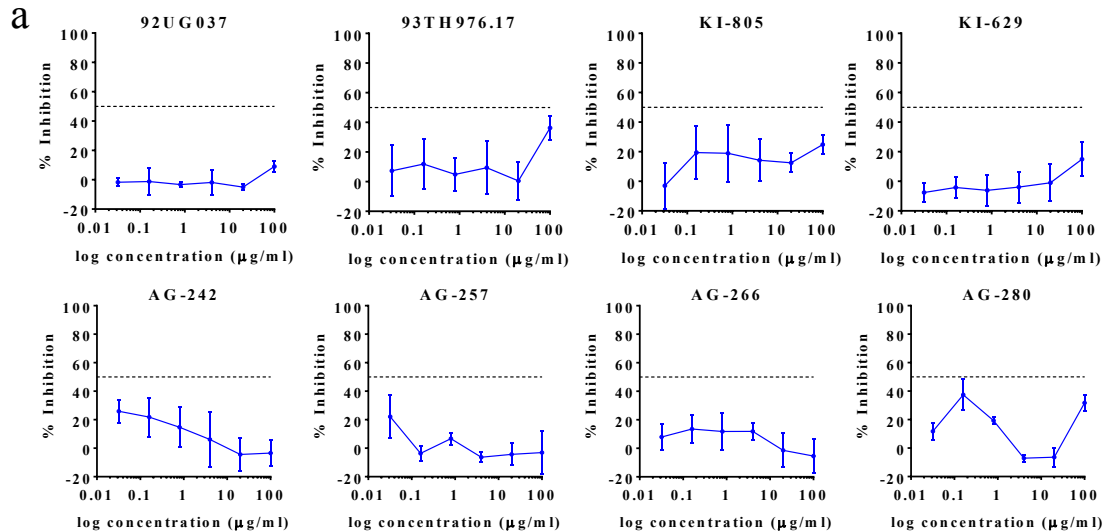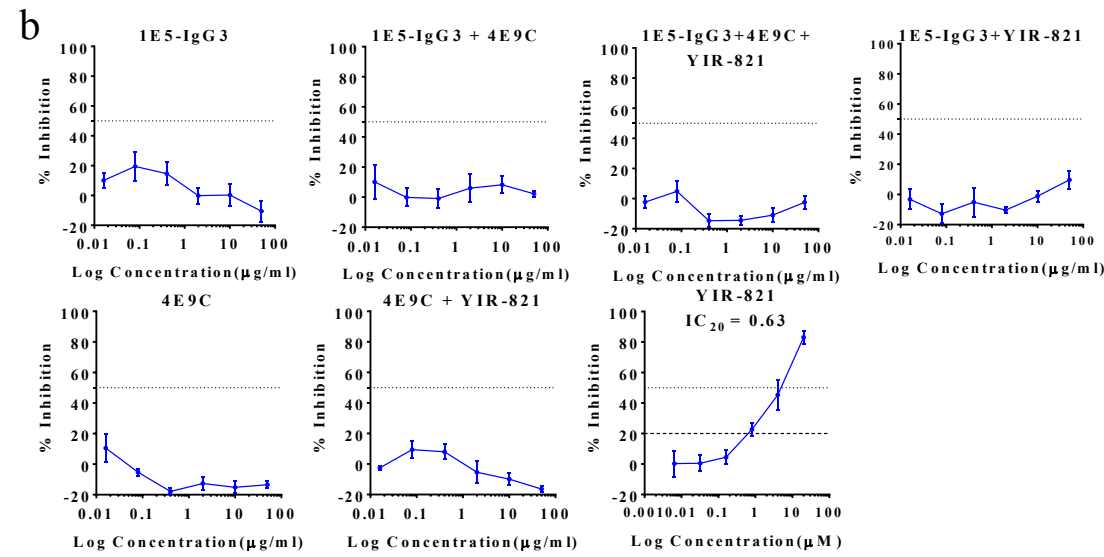

Supplement: Supplementary file 6 — Additional file 6: Fig. S6. 1E5 is a non-neutralizing antibody. (a) The neutralization activity of 1E5-IgG1 was tested in TZM-bl assays against Env-pseudotyped viruses from subtype A, CRF01_AE and CRF02_AG of HIV-1. (b) Neutralization activity of 1E5-IgG3 was examined alone or with anti-CoRBS Ab (4E9C) and CD4mc (YIR-821) against pseudotyped virus with AG-257 Env. [file 12977_2021_568_MOESM6_ESM.pdf]

— NHG      — Test IgG      — Test IgG + YIR-821  
— Test IgG + YIR-821 + 17b      — Test IgG + YIR-821 + 4E9C

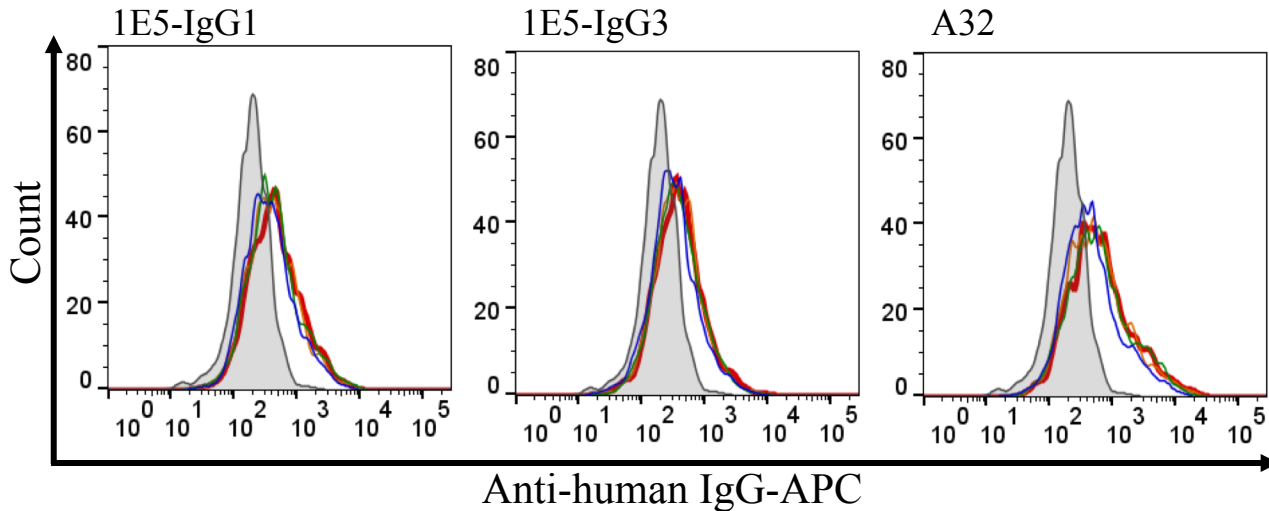

Supplement: Supplementary file 7 — Additional file 7: Fig. S7. Anti-CoRBS antibodies failed to increase the binding of anti-cluster A antibodies to AG-252 Env. HEK 293T cells expressing both EGFP and AG-252 Env were stained with biotinylated test IgG (1E5-IgG1, 1E5-IgG3 and A32 at 10 µg/ml) alone or with anti-CoRBS antibody (17b or 4E9C, 5 µg/ml) and CD4mc YIR-821 (20 µM), and the binding was detected by APC-conjugated streptavidin. Histograms of APC in EGFP+ cells are shown. [file 12977_2021_568_MOESM7_ESM.pdf]

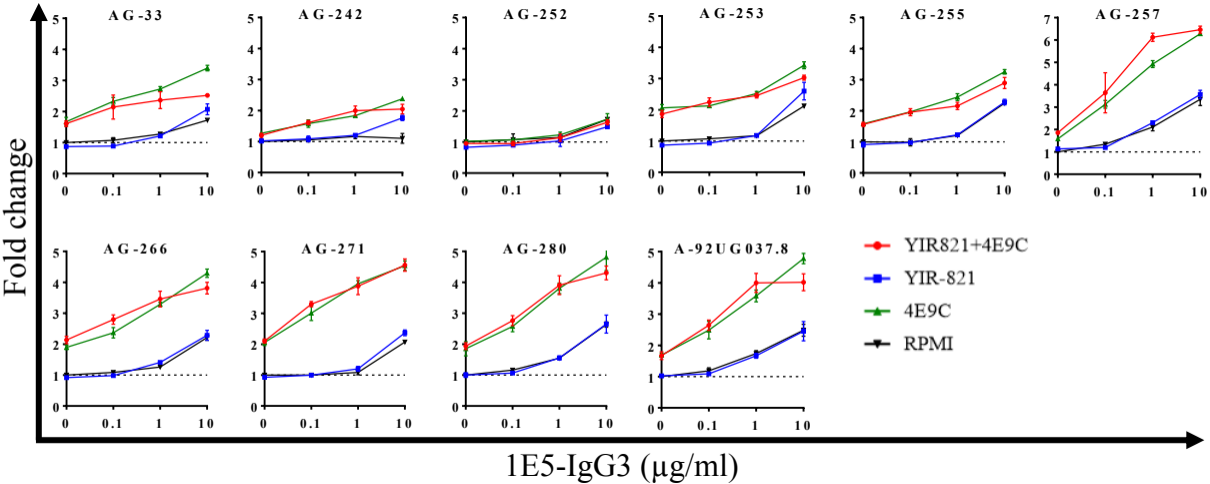

Supplement: Supplementary file 8 — Additional file 8: Fig. S8. IgG3 form of 1E5 shows stronger ADCC in the presence of anti-CoRBS antibody. The effect of anti-CoRBS antibody and CD4mc on ADCC activity, which was measured by signaling through FcγRIIIa, was analyzed using IgG3 forms of 1E5. HEK 293T cells transfected with plasmid expressing HIV-1 Env (nine binding assay-positive Env proteins from a CRF02_AG panel and one subtype A) were incubated with the indicated antibodies and co-cultivated with the ADCC indicator cell line expressing FcγRIIIa. ADCC activities obtained by serial dilution of 1E5 (0.1, 1 and 10 µg/ml) alone (RPMI) or in combination with 4E9C (5 μg/ml) and YIR-821 (20 µM) are shown. ‘0’ on the X axis indicates the absence of 1E5. The fold change was calculated by dividing the luminescence units in the presence of Ab by those in the absence of Ab. Experiments were performed in triplicate, and the means ± standard errors of the means are shown. [file 12977_2021_568_MOESM8_ESM.pdf]

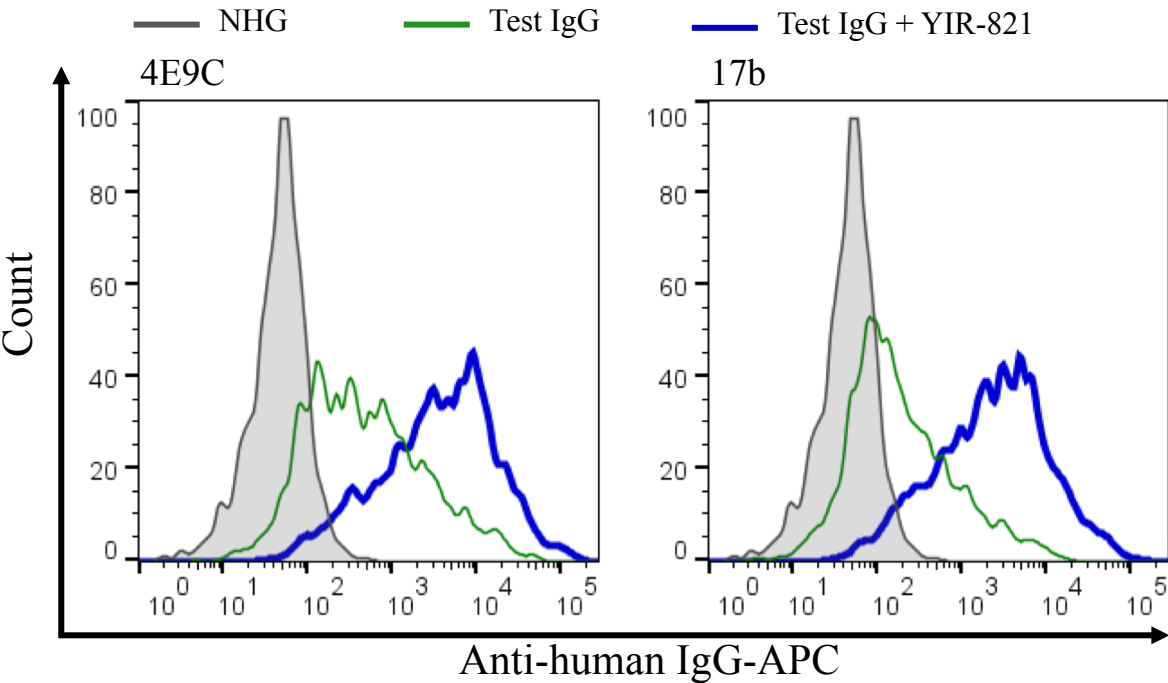

Supplement: Supplementary file 9 — Additional file 9: Fig. S9. Recognition of target cells by anti-CoRBS antibodies. HEK 293T cells expressing both EGFP and AG-257 Env were stained with test IgG (4E9C and 17b, 10 µg/ml) alone or in the presence of CD4mc YIR-821 (20 µM). Antibody binding was detected by APC-conjugated anti-human IgG secondary Ab. Histograms of APC in EGFP+ cells are shown [file 12977_2021_568_MOESM9_ESM.pdf]
