# Supplementary material for: Functional analysis of a monoclonal antibody reactive against the C1C2 of Env obtained from a patient infected with HIV-1 CRF02_AG
Source: Retrovirology. 2021 Aug 21;18:23. doi: 10.1186/s12977-021-00568-y (PMC8379604; doi:10.1186/s12977-021-00568-y)
Supplement: Supplementary file 10 — Additional file 10: Table S1. Second-round PCR primers. [file 12977_2021_568_MOESM10_ESM.pdf]

Table S1. Second-round PCR primers

Primers	5´-3´ Sequence
**Forward Primers**	
**VH**	
5' SgrAI VH1	TGGTGGCAGCAGCCAccGGTGTACATTCCCAGGTGCAGCTGGTGCAG
5' SgrAI VH1-5	TGGTGGCAGCAGCCAccGGTGTACATTCCGAGGTGCAGCTGGTGCAG
5' SgrAI VH3	TGGTGGCAGCAGCCAccGGTGTACATTCTGAGGTGCAGCTGGTGGAG
5' SgrAI VH3-23	TGGTGGCAGCAGCCAccGGTGTACATTCTGAGGTGCAGCTGTTGGAG
5' SgrAI VH4	TGGTGGCAGCAGCCAccGGTGTACATTCCCAGGTGCAGCTGCAGGAG
5' SgrAI VH4-34	TGGTGGCAGCAGCCAccGGTGTACATTCCCAGGTGCAGCTACAGCAGTG
5' SgrAI VH1-18	TGGTGGCAGCAGCCAccGGTGTACATTCCCAGGTTCAGCTGGTGCAG
5' SgrAI VH1-24	TGGTGGCAGCAGCCAccGGTGTACATTCCCAGGTCCAGCTGGTACAG
5' SgrAI VH3-33	TGGTGGCAGCAGCCAccGGTGTACATTCTCAGGTGCAGCTGGTGGAG
5' SgrAI VH3-9	TGGTGGCAGCAGCCAccGGTGTACATTCTGAAGTGCAGCTGGTGGAG
5' SgrAI VH4-39	TGGTGGCAGCAGCCAccGGTGTACATTCCCAGCTGCAGCTGCAGGAG
5' SgrAI VH6-1	TGGTGGCAGCAGCCAccGGTGTACATTCCCAGGTACAGCTGCAGCAG
**VK**	
5' EcoRV Vk 1-5	GGTGCCTACGGGGATATCCAGATGACCCAGTC
5' EcoRV Vk 1-9	GGTGCCTACGGGGATATCCAGTTGACCCAGTCT
5' EcoRV Vk 1D-43	GGTGCCTACGGGGATATCCGGATGACCCAGTC
5' EcoRV Vk 2-24	GGTGCCTACGGGGATATTGTGATGACCCAGAC
5' EcoRV Vk 2-28	GGTGCCTACGGGGATATTGTGATGACTCAGTC
5' EcoRV Vk 2-30	GGTGCCTACGGGGATGTTGTGATGACTCAGTC
5' EcoRV Vk 3-11	GGTGCCTACGGGGATATTGTGTTGACACAGTC
5' EcoRV Vk 3-15	GGTGCCTACGGGGATATAGTGATGACGCAGTC
5' EcoRV Vk 3-20	GGTGCCTACGGGGATATTGTGTTGACGCAGTCT
5' EcoRV Vk 4-1	GGTGCCTACGGGGATATCGTGATGACCCAGTC
**VL**	
5' SfoI Vl1	ACTTTCTGCACAGGCTCCTGGGCCCAGTCTGTGCTGACKCAG
5' SfoI Vl2	ACTTTCTGCACAGGCTCCTGGGCCCAGTCTGCCCTGACTCAG
5' SfoI Vl3	ACTTTCTGCACAGGCTCTGTGACCTCCTATGAGCTGACWCAG
5' SfoI Vl4/5	ACTTTCTGCACAGGCTCTCTCTCSCAGCYTGTGCTGACTCA
5' SfoI Vl6	ACTTTCTGCACAGGCTCTTGGGCCAATTTTATGCTGACTCAG
5' SfoI Vl7/8	ACTTTCTGCACAGGCTCCAATTCYCAGRCTGTGGTGACYCAG
**Reverse primers**	
**VH**	
APS-R	GGGGGAAGACCGATgggc
**VK**	
HCK5-B	GAAGACAGATGGTGCAGCCACAGT
**VL**	
VL-R	CAGTGTGGCCTTGTTGGCTTG
